# ‘Carbon-Monoxide-Releasing Molecule-2 (CORM-2)’ Is a Misnomer: Ruthenium Toxicity, Not CO Release, Accounts for Its Antimicrobial Effects

**DOI:** 10.3390/antiox10060915

**Published:** 2021-06-05

**Authors:** Hannah M. Southam, Michael P. Williamson, Jonathan A. Chapman, Rhiannon L. Lyon, Clare R. Trevitt, Peter J. F. Henderson, Robert K. Poole

**Affiliations:** 1 Department of Molecular Biology and Biotechnology, The University of Sheffield, Western Bank, Sheffield S10 2TN, UK; hannahmsoutham@gmail.com (H.M.S.); m.williamson@sheffield.ac.uk (M.P.W.); J.A.Chapman2@newcastle.ac.uk (J.A.C.); rhiannon.lyon@sky.com (R.L.L.); clare.trevitt@gmail.com (C.R.T.); 2 Centre for Bacterial Cell Biology, Medical School, Newcastle University, Newcastle upon Tyne NE2 4AX, UK; 3 School of Biomedical Sciences and Astbury Centre for Structural Molecular Biology, University of Leeds, Leeds LS2 9JT, UK; p.j.f.henderson@leeds.ac.uk

**Keywords:** amino acid, antimicrobial agent, bacteria, carbon monoxide, CORM-2, glutathione, metal, ruthenium compound, thiol

## Abstract

Carbon monoxide (CO)-releasing molecules (CORMs) are used to deliver CO, a biological ‘gasotransmitter’, in biological chemistry and biomedicine. CORMs kill bacteria in culture and in animal models, but are reportedly benign towards mammalian cells. CORM-2 (tricarbonyldichlororuthenium(II) dimer, Ru_2_Cl_4_(CO)_6_), the first widely used and commercially available CORM, displays numerous pharmacological, biochemical and microbiological activities, generally attributed to CO release. Here, we investigate the basis of its potent antibacterial activity against *Escherichia coli* and demonstrate, using three globin CO sensors, that CORM-2 releases negligible CO (<0.1 mol CO per mol CORM-2). A strong negative correlation between viability and cellular ruthenium accumulation implies that ruthenium toxicity underlies biocidal activity. Exogenous amino acids and thiols (especially cysteine, glutathione and N-acetyl cysteine) protected bacteria against inhibition of growth by CORM-2. Bacteria treated with 30 μM CORM-2, with added cysteine and histidine, exhibited no significant loss of viability, but were killed in the absence of these amino acids. Their prevention of toxicity correlates with their CORM-2-binding affinities (Cys, *K*_d_ 3 μM; His, *K*_d_ 130 μM) as determined by ^1^H-NMR. Glutathione is proposed to be an important intracellular target of CORM-2, with CORM-2 having a much higher affinity for reduced glutathione (GSH) than oxidised glutathione (GSSG) (GSH, *K*_d_ 2 μM; GSSG, *K*_d_ 25,000 μM). The toxicity of low, but potent, levels (15 μM) of CORM-2 was accompanied by cell lysis, as judged by the release of cytoplasmic ATP pools. The biological effects of CORM-2 and related CORMs, and the design of biological experiments, must be re-examined in the light of these data.

## 1. Introduction

Carbon monoxide (CO) has been a valuable tool in biological chemistry since the 1890s when Hoppe-Seyler demonstrated the absorbance spectrum of CO-haemoglobin in pigeon heart muscle. CO continues to be used as a haem ligand [1] and is treated with respect in the laboratory owing to its well-known toxicity. Nevertheless, CO is produced endogenously in biology by haem oxygenases (HO) and its biological functions are actively studied [2]. The gas is now recognised as a potent biological messenger in mammalian systems and is involved in diverse signalling processes, including the production of inflammatory mediators [3], cell survival and apoptosis [4], signalling in the central nervous system [5] and in bacterial infection [6].

Over the past decade, many novel transition metal-based carbon monoxide-releasing molecules (CORMs) have been developed to mimic haem oxygenase activity and deliver physiologically relevant levels of CO experimentally or therapeutically [7,8]. The widespread use of these compounds as CO donors has accelerated our understanding of CO as an important gasotransmitter molecule in human and animal biology [9]. Increasingly, CORMs are considered as potential pro-drugs for CO delivery, not only in clinical and physiological applications [10,11], but also as anticancer drugs [12] and antimicrobials [13]. Recently, hybrid CORMs have been developed in which, for example, a cobalt–carbonyl as a CO releaser is complexed with a fumaric acid derivative as an Nrf2/HO-1 activator [14]. Thus, CORM biology is a highly active area of research.

The earliest report that CORMs liberate CO describes CORM-2 (Ru_2_Cl_4_(CO)_6_, tricarbonyldichlororuthenium(II) dimer) [15], which is readily available commercially at low cost. More than 400 papers now describe its activities and uses. Spectrophotometric and NMR analysis revealed that it releases CO in a concentration-dependent manner and caused sustained vasodilation in pre-contracted rat aortic rings, attenuated coronary vasoconstriction in hearts ex vivo and significantly reduced acute hypertension in vivo. These vascular effects were mimicked by induction of HO-1 after treatment of animals with haemin, which increases endogenously generated CO [15]. Injection of CORM-2 into mice increased phagocytosis of bacteria and rescued haem oxygenase-deficient mice from sepsis-induced lethality [16]. In the light of the above controls and the use of haemoglobin in bacterial cultures to scavenge released CO [17], it has been tacitly assumed that all experimental effects of CORM-2 (and other CORMs) are due to the released CO. For example, a recent study demonstrated the significant enhancement by CORM-2 of clot formation and strength in the plasma of dogs, and this was attributed to the released CO [18]. Gastric ulcer healing in rats was also accelerated by CORM-2 modulated by increased gastric mucosal content of CO released from its pharmacological donor; CO content in gastric mucosa was elevated after CORM-2 administration [19]. In some studies, however, CO gas and CORM-2 are erroneously assumed to be equivalent: for example, Zacharia et al. [20] isolated CO-resistant mutants of *Mycobacterium tuberculosis*, in a gene named *cor* (CO resistance), after growth of a transposon library in the presence of 2% CO gas, but the susceptibility to CO of *E. coli* cells expressing the *cor* gene was evaluated with CORM-2.

**Figure 1:**
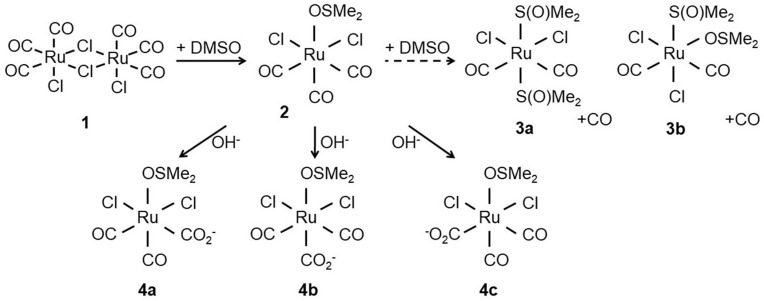
Structure of CORM-2 (**1**) prior to dissolution in dimethyl sulfoxide (DMSO). After addition of DMSO, the chloride bridges of the CORM-2 dimer are displaced by attack of DMSO to the Ru(II) ion(s) yielding two identical [RuCl_2_(CO)_3_DMSO] monomers (**2**). Next, **2** further reacts with DMSO to yield [RuCl_2_(CO)_2_-(DMSO)] isomers (**3a**,**3b**) with concomitant release of CO. Alternatively, the carbonyl ligands can react with water to give carboxylates (**4a**–**c**). Adapted from [24].

Despite its widespread use, CORM-2 has the serious disadvantage of being insoluble in water, aqueous media and many organic solvents. For biological use, CORM-2 is dissolved in dimethylsulfoxide (DMSO) where it undergoes [21,22] a transition into mononuclear Ru(II)–carbonyl complexes by displacement of the chloride bridges with DMSO [21,23,24], but there are additional complex changes that are incompletely understood. These monomers may react further with DMSO, via displacement of one CO ligand by coordination of the sulphur atom of DMSO to the Ru(II) ion, yielding [RuCl_2_(CO)_2_-(DMSO)_2_] isomers, which are relatively stable. As a consequence of this chemistry, CORM-2 solutions in DMSO probably represent a mixture of the forms in Figure 1, depending on the age of the solution [21,22]. Such heterogeneous mixtures may undergo further chemical changes and ligand exchange. The availability of CO is of paramount importance. Desmard [25] classified CORM-2 as a ‘fast CO releaser’, based on its ability to rapidly convert ferrous myoglobin to carbonmonoxy myoglobin in the presence of dithionite. However, little or no CO was detected on addition of, presumably heterogeneous, stock solutions of CORM-2 to buffers or media when assayed via the alternative oxyhaemoglobin assay (which avoids dithionite [26]), or by a CO electrode, gas-phase Fourier transform infrared spectroscopy or gas chromatography [17,21,22,25].

We recently proposed a radically different explanation for the toxic biological activities of a related Ru–carbonyl CORM, namely CORM-3, [Ru(CO)_3_Cl(glycinate)], and argued that, rather than acting as an antimicrobial agent via release of CO, CORM-3 is a source of Ru(II), which reacts with cellular targets [27]. Indeed, over 200 publications report the antimicrobial activities of various Ru-based compounds that are not CORMs; in some, the Ru ions play a direct functional role, directly coordinating to biological targets [24]. These findings have far-reaching implications for understanding the toxicity of these agents against both microbial and mammalian cells and for their possible development as useful pharmaceuticals. 

However, since CORM-2, not CORM-3, is by far the pre-eminent and commercially affordable CORM for biological research, we describe now new studies of its CO release in relation to its antimicrobial potential and of its interactions with amino acids and metabolic thiols: we conclude that the term ‘CO-releasing molecule’ is, in many experimental designs, a misnomer. 

## 2. Materials and Methods

### 2.1. Preparation of CORM-2 Stocks

Unless otherwise stated, all chemicals and reagents were purchased from Sigma-Aldrich (Poole, UK). CORM-2 dimer Ru_2_Cl_4_(CO)_6_ (tricarbonyldichlororuthenium(II)) was purchased from Sigma-Aldrich (Poole, UK). and dissolved in dimethyl sulfoxide (DMSO) to final concentrations of 10–40 mM. Stocks were further diluted to <10 mM with distilled H_2_O. CORM-2 solutions were shielded from light and, with the exception of intentionally prolonged CO-release experiments (described below), used within 10–15 min of dissolution. For assessment of the effects of growth media on CORM-2 antimicrobial activity, 10 mM CORM-2 stocks were prepared in DMSO and then diluted 10-fold with either sterile H_2_O (as standard), 10 mM phosphate-buffered saline (PBS), glucose-defined minimal medium (GDMM), Luria broth (LB), Mueller Hinton II broth (MH-II), Dulbecco’s modified Eagle medium (DMEM) or Roswell Park Memorial Institute medium (RPMI) as described before [28]. The resulting CORM/media solutions were incubated at room temperature for 10 min and added to bacterial cultures to a final concentration of 30 µM CORM-2 and 5% (*v*/*v*) medium, meaning that the DMSO concentration was generally 0.3% in these experiments. To study the effects of exogenous amino acids, sulphur compounds or nucleotide monophosphates on CORM-2 antimicrobial activity, the procedure employed was as previously described [27]. Briefly, 10–40 mM CORM-2 stocks, prepared in DMSO, were incubated with a 2-fold excess of individual amino acids, sulfur compounds (glutathione (GSH), oxidised glutathione (GSSG), sodium hydrosulphide, N-acetyl-cysteine (NAC)) or nucleotide monophosphates (AMP, CMP, GMP or TMP). The resulting solutions were incubated at room temperature prior to addition to bacterial cultures to a final concentration of 30 µM CORM-2 (maximum 0.3% DMSO) and 60 µM amino acid, sulphur compound or nucleotide monophosphate.

### 2.2. CO-Release Measurements (Oxyhaemoglobin and Oxyneuroglobin Assays)

**Figure 2:**
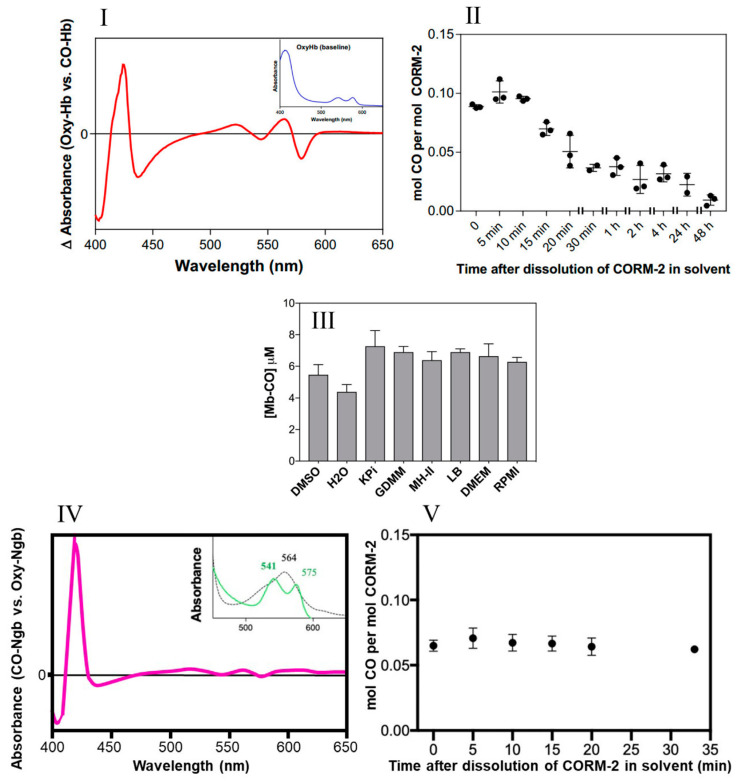
Little CO is released from CORM-2 in DMSO**.** (**I**) CO-difference UV-visible spectrum of CO-saturated haemoglobin with the absolute spectrum of the oxyHb sample (shown in inset) as a baseline. (**II**) Amount of CO detected as COHb expressed as a function of the age of the CORM-2 stock solution, i.e., time after dissolution of CORM-2 in DMSO (solvent). (**III**) Maximum yields of Mb-CO from 10 μM CORM-2 as an average of two technical repeats, with SD shown as error bars. CO was detected after prior incubation for 20 min in a 10-fold excess of DMSO, water, KPi, GDMM, MH-II, LB, DMEM or RPMI. (**IV**) CO-difference UV-visible spectrum of neuroglobin with saturating CORM-2. The inset shows the absolute spectra of the reduced (dotted) and CO-bound forms (green). (**V**) Amount of CO detected as CO-Ngb expressed as a function of the age of the CORM-2 stock solution, i.e., time after dissolution of CORM-2 in DMSO. Data in **II** and **V** display individual data points from 3 independent replicates. Minimal autoxidation of the oxyglobins was established prior to each time point in **II** and **V**.

Measurements of CO release from CORM-2 stock solutions were conducted via oxyhaemoglobin assays as outlined before [26]. Briefly, bovine haemoglobin (Hb) in 0.1 M KPi buffer pH 7.4 was reduced by the addition of excess sodium dithionite and then desalted on a PD-25 column (GE Healthcare, Little Chalfont, UK) to obtain oxyhaemoglobin (oxyHb). Release of CO from CORM-2 was observed by changes in the Soret region (~422 nm) of the oxyHb visible absorbance spectrum following displacement of the bound O_2_ by CO to yield carbonmonoxy-haemoglobin (COHb). Stock solutions of CORM-2 (10 mM) were prepared in DMSO and then added to ~12–13 µM oxyHb to a final concentration of 100 µM CORM-2. The solutions were then incubated for 2 min and CO-difference spectra were measured using the spectrum of the oxyHb sample as a baseline. Essentially identical methods were used with purified neuroglobin [29], kindly provided by Dr Jesus Tejero, University of Pittsburgh. Prior to each CORM-2 addition at the ages after DMSO dissolution shown in Figure 2, the level of autoxidation of the oxygenated form was checked in visible spectra and found to be insignificant. It is important to note that, under our conditions, CO released from CORM-2 never saturated the oxygenated globins used as sensors. The amount of carbonmonoxy globin in the samples resulting from CO released from CORM-2 was then quantified from published extinction coefficients and comparison with the CO-difference spectra of the sample obtained by saturation with CO gas for 2 min. Between time points, CORM-2 stocks were stored shielded from light and were mixed prior to addition to oxyHb.

### 2.3. General Bacterial Methods and Strains

Bacterial strains employed in this study were *E. coli* K-12 strain MG1655 (F^−^ lambda^−^ *ilvG*, *rfb-50*, *rph-1*) and a GSH-deficient derivative strain (*E. coli* MG1655 *gshA*) which contained a kanamycin resistance cassette in place of the gene for *gshA* encoding a γ-glutamate-cysteine ligase. Correct insertion of the Kan^R^ cassette was confirmed by PCR and measurements of total cellular thiol content via 5,5-dithio-bis-(2-nitrobenzoic acid) (DTNB) assays of cell-free extracts, essentially as described before [30]. Growth of *E. coli* cultures was assessed via standard methods using either optical density measurements in a Jenway 7305 spectrophotometer (Felstead, UK) at 600 nm or in a Tecan Sunrise Plate reader (Thermo Fisher Scientific, Horsham, UK). Viability counts were made using the Miles–Misra method [31]. Strains were routinely grown aerobically at 37 °C in the phosphate salts-based glucose-defined minimal medium (GDMM) [32].

### 2.4. Assessment of CORM-2 Accumulation and Subcellular Distribution

CORM-2 accumulation by *E. coli* cells was determined by measuring Ru content of *E. coli* cell pellets following exposure to CORM-2 by inductively-coupled plasma-atomic emission spectroscopy (ICP-AES), as described previously [27,33]. We used washes of 0.5% nitric acid to remove extraneous metal [34], but it is not possible to discriminate unambiguously between loosely bound and tightly bound metal. To determine subcellular localisation of CORM-derived Ru, early exponential phase *E. coli* cultures were treated with 30 µM CORM-2 for 1 h and then harvested by centrifugation at 5000× *g* for 20 min at 4 °C. Supernatant samples containing excess CORM-2 were discarded. Pellets were washed in ice-cold phosphate-buffered saline to remove loosely associated CORM-2 and a fraction was retained for determination of Ru content of the whole cellular fraction. Next, cell pellets were resuspended in 50 mM Tris-HCl (pH 8.0) and broken by sonication. Unbroken cells/debris were removed by a low-spin centrifugation (15,000× *g*) to obtain cell lysates. The unbroken cells and debris were analysed by ICP-AES for the ‘debris’ fraction. Cell lysates were then subjected to a high-spin ultracentrifugation (215,000× *g* for 1 h at 4 °C) to obtain an insoluble membrane pellet (containing both inner and outer membranes) and a soluble cytoplasmic/periplasmic fraction. Each fraction was prepared for ICP-AES as described before [27,33]. For determination of Ru content of *E. coli* genomic DNA, cell cultures were treated with 30 µM CORM-2 as described above, washed to remove excess CORM-2 and then genomic DNA was extracted using the Wizard Genomic DNA Extraction Kit (Promega, Southampton, UK) as per manufacturer’s instructions. DNA was quantified via a NanoDrop ND-1000 spectrophotometer (Thermo Fisher Scientific, Horsham, UK) and then genomic DNA samples were analysed for Ru content by ICP-AES.

### 2.5. Interactions of CORM-2 with Amino Acids or Glutathione Via ^1^H NMR

Synthetic peptides (Genscript, Leiden, Netherlands) A_3_CA_3_, A_3_DA_3_, A_3_HA_3_, A_3_MA_3_ and A_7_ had N-terminal acetylation and C-terminal amidation. ^1^H NMR experiments were conducted in 30 mM KPi buffer prepared in distilled H_2_O at pH 7.4 then freeze-dried and re-dissolved in deuterated water (D_2_O) prior to experiments. Stock solutions of peptides were generally prepared in 30 mM KPi buffer pH 7.4 but peptides with low solubility were dissolved in deuterated DMSO (d^6^-DMSO) and further diluted with buffer. A maximum of 10% (*v*/*v*) d_6_-DMSO was present during titration experiments. CORM-2 stocks were prepared in d_6_-DMSO and then further diluted 10-fold in D_2_O and typically used within 1–2 h. Peptide concentrations were determined by ^1^H NMR relative to the internal standard trimethylsilylpropionate (TSP, 100 μM) using a long recycle time. Titrations with reduced glutathione (GSH) or oxidised glutathione (GSSG) were conducted as for synthetic peptides. For titrations, peptide or glutathione solutions were prepared and quantified prior to the addition of CORM-2 or the equivalent level of d_6_-DMSO as a control for the solvent. ^1^H NMR experiments were carried out on a Bruker Avance-1 800 MHz spectrometer (Coventry, UK) using pre-saturation for solvent suppression. Baselines were corrected manually before spectral signals were integrated using Bruker Topspin software version 4.0.5 (Coventry UK). Estimations for CORM peptide-binding affinities, expressed as the dissociation constant, *K*_d_, were determined as described [27,35], based either on integrated peak intensity (slow exchange conditions) or on chemical shift (fast exchange conditions), using the following equation:

Δδ_obs_ = Δδ_max_ [([*P*]_t_ + [*L*]_t_ + *K*_d_) − [([*P*]_t_ + [*L*]_t_ + *K*_d_)^2^ − 4[*P*]_t_ [*L*]_t_]^½^]/2[*P*]_t_ (1)

where: Δδ_obs_ is the change in the observed shift from the free state and Δδ_max_ is the maximum shift change in saturation, and [*P*] and [L] represent the concentrations of free protein and free ligand. *K*_d_ is the concentration of ligand and protein required to saturate half the binding sites.

### 2.6. ATP Release Assays

The levels of extracellular ATP in *E. coli* cultures grown with or without CORM-2 were determined as follows. Culture samples were rapidly harvested by brief centrifugation at 15,000× *g* at 4 °C to remove cells. Supernatants were retained at −20 °C. ATP analysis of the extracellular supernatants was conducted using the bioluminescence-based Molecular Probes ATP Determination Kit (Invitrogen, Thermo Fisher Scientific, Horsham, UK). The levels of ATP in the supernatants were derived via a standard curve of ATP solutions from 1 nM–1 µM. Luminescence measurements were measured in duplicate on a Lumat^3^ Luminometer (Berthold Technologies, Harpenden, UK).

### 2.7. Data and Statistical Analysis

All biological experiments were conducted with a minimum of three biological repeats and often 2 or more technical repeats. General data handling and determination of means and standard deviations were performed in Microsoft Excel. Statistical analyses (ANOVAs, Tukey’s multiple comparisons tests, *t*-tests, Pearson’s correlational analysis) and the fitting of data to standard curves were conducted in GraphPad Prism Software. 

## 3. Results

### 3.1. CORM-2 Releases Negligible Amounts of CO in Biological Experiments

Unlike CORM-3, which is water-soluble, CORM-2 requires solubilisation in DMSO where it undergoes extensive ligand exchange with the solvent and isomerises with implications for CO release (Figure 1). CO is liberated by ligand substitution by DMSO in steps **3a** and **3b** (Figure 1) but the stoichiometry between **2**, **3a** and **3b** is uncertain [21,22]; species **4a**, **4b** and **4c** show possible further reactions with water to form carboxylates. Thus the complexity of the dissolution of CORM-2 in DMSO frustrates understanding the amount of biologically accessible CO and is likely to depend on the age of the stock solution. 

The classification [25] of CORM-2 as a ‘fast CO releaser’ was based on its ability to convert ferrous myoglobin to carbonmonoxy myoglobin. This assay requires sodium dithionite to reduce myoglobin, but dithionite (and also sodium sulphite and potassium metabisulphite) facilitate the release of CO [26]. Here, we used three different globins to investigate CO release and inform our studies of the mechanism of CORM-2 toxicity. 

To investigate CO release from CORM-2 in DMSO, but avoid dithionite, we measured CO release from CORM-2 at various intervals after dissolution in DMSO by conversion of two oxygenated globins—oxyhaemoglobin (oxyHb) and oxyneuroglobin (oxyNgb)—into the respective carbonmonoxy forms; these assays obviate the need for dithionite because, unlike myoglobin, these globins bind CO with much greater affinities than they bind oxygen [29,36], and so it is not necessary to deoxygenate reduced globin solutions to measure the CO release kinetics of CORMs. 

The amount of CO liberated was quantified via the optical changes that occur as CO displaces O_2_ bound to the globins after addition of CORM-2 (26). The spectral features of the reaction of haemoglobin with CO in saturated solutions are shown in Figure 2I. Figure 2II shows the use of this assay to determine the level of CO released from CORM-2 stocks at intervals of ‘aging’ after dissolution in DMSO. Detectable CO, initially approx. 10% of the available CO, decreased rapidly over time (Figure 2II); less than 0.05 mol CO per CORM-2 dimer was measured after 20 min. Strikingly, the maximum level of CO released from CORM-2 was approximately 0.1 mol CO per mol CORM-2, occurring at 5–10 min after dissolution in DMSO. 

Second, to assess whether the use of various growth media in future experiments might interfere with CO release, we measured the amount of CO generated by CORM-2 via the widely used assay involving conversion of deoxymyoglobin (Mb) to carbonmonoxymyoglobin (MbCO) in the presence of dithionite to promote CO release [26]. Figure 2III shows that the level of CO released from CORM-2 following pre-incubation in DMSO or water was 0.44–0.55 equivalents. There were no significant differences observed when CORM-2 was preincubated with phosphate buffer (KPi), a defined growth medium (GDMM), or any of a range of complex growth media (see Figure 2III). Therefore, we conclude that these rich nutrient media do not interfere with CO release (see later).

Human haemoglobin has an affinity for CO approximately 200–250 times greater than for oxygen [36,37]. Nevertheless, to assess the potential availability in vivo of a presumptive higher-affinity site for binding of CO from CORM-2, we used neuroglobin (Ngb), which is expressed in brain and retina and has an affinity for CO 500 times higher than haemoglobin. The distinct optical changes that occur on reaction of oxyneuroglobin with CO (Figure 2IV) allowed confirmation of the level of CO released from CORM-2 on ‘aging’ in DMSO. The highest occupancy of CO was at 5 min after preparation of the CORM-2 (Figure 2V) and accounted for about 7% of the available CO if one CO were released in stages 3a and 3b of Figure 1. Thus, in the experiments conducted in this study, without dithionite, we estimate that only a maximum of ~0.07 to 0.1 mol CO per CORM-2 was present during the microbiological studies. Thus, in experiments using CORM-2 as a source of CO, the time interval between dissolution in DMSO and experimental use should be as short as possible and no longer than about 10–15 min, to avoid even lower levels of available CO.

Although it would be desirable to measure independently the *K*_d_ of CORM-2 for CO, this would be extremely problematic. As shown above, the CORM-2 dimer, when dissolved, is solvated to break into monomers. It would be difficult to resolve the kinetics of CO release and rebinding. When CORM-2 is dissolved in water, CO_2_ is formed, giving an additional complication [22]. Finally, in water the Cl^−^ is replaced by water. We therefore believe that useful kinetic studies are fraught with difficulties and they were not attempted.

### 3.2. CORM-2 Exhibits Ru-Related Antimicrobial Activity Against Escherichia coli

**Figure 3:**
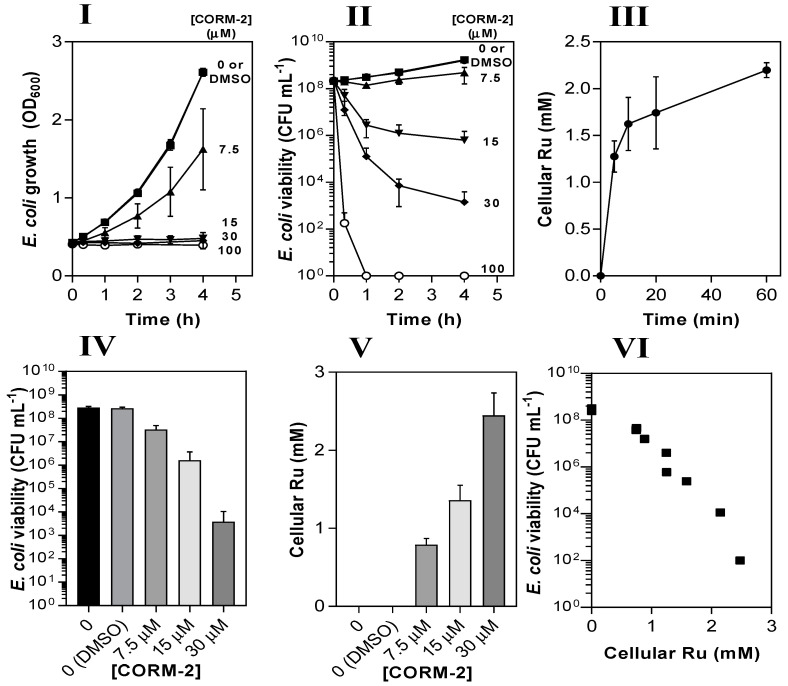
The antimicrobial activity of CORM-2 against *E. coli* is strongly correlated with intracellular Ru accumulation. The dose-dependent inhibitory effects of CORM-2 (0–100 µM) on *E. coli* growth (**I**) and viability (**II**) as determined by measuring optical density (OD_600_) and colony-forming units (CFU). Uptake of CORM-2 (**III**) was assessed by measuring cellular Ru content at time intervals after addition of 30 µM CORM to early log-phase *E. coli*. The viability (**IV**) of *E. coli* cultures 1 h after addition of 0–30 µM CORM-2 was compared to the level of intracellular Ru (**V**). The data in **IV** and **V** are plotted in **VI** to show the negative correlation between the culture viability and the extent of Ru accumulation (r^2^ = 0.92). Cells were grown on GDMM in all experiments. Cellular Ru content was assessed by conducting ICP-AES on culture samples. Data represent three biological repeats ± standard deviation (SD) and the data in (**VI**) were analysed via a Pearson’s (two-tailed) correlational analysis (*p* ≤ 0.0001).

CORM-2 caused dose-dependent (7.5–100 µM) inhibition of growth of *E. coli* MG1655 (Figure 3I) grown aerobically on minimal salts-based medium (glucose-defined minimal medium, GDMM). Based on data in Figure 2, the amount of CO released was estimated to be <10 µM (corresponding to 10% of the CORM-2 concentration after 4 h growth), much too low to have significant toxic effects [38]. Bactericidal effects were observed at 15–100 µM CORM-2 (Figure 3II). Thus, subsequent experiments in this study focused on the role of the Ru(II) ions of CORM-2.

Next, we measured the Ru contents of *E. coli* cell pellets via inductively coupled plasma-absorption emission spectroscopy (ICP-AES) at time intervals after addition of 30 µM CORM-2 (Figure 3III). Initial uptake of Ru by *E. coli* cells occurred at ~250 µM Ru min^−1^ and reached a final intracellular concentration of ~2 mM after 60 min, approx. 60-fold higher than extracellular concentrations. To determine whether cellular accumulation of Ru was related to loss of culture viability, cell cultures were incubated for 1 h with 7.5–30 µM CORM-2 and then corresponding samples were tested for viability (Figure 3IV) and cellular Ru content (Figure 3V). There was a strong negative correlation between the viability of cell cultures and the extent of cellular Ru accumulation (r^2^ = 0.92), implying that the antimicrobial effects of CORM-2 are linked to the accumulation of Ru (Figure 3VI). 

A fraction of the accumulation of Ru observed in Figure 3 might be accounted for by adsorption to the surface of the cells. This confounds virtually all studies of metal compound uptake by bacteria because of the net negative charge of the surface layers. To mitigate this, we washed cells with nitric acid but cannot unambiguously distinguish between loosely bound and tightly bound metal. Nevertheless, in a test of the efficacy of 1% nitric acid in removing loosely bound Ru, we found no significant difference between the Ru content of cells washed three times in acid or once in 10 mM K phosphate buffer (results not shown). Although the relatively basic subcellular fractionation procedures used may include externally exposed layers in the membrane and debris fractions, the appearance of Ru in extracted DNA (see later) demonstrates that Ru does enter cells. Thus, much of the accumulated ruthenium in the ‘soluble’ fraction is probably bound to DNA.

### 3.3. Exogenous Amino Acids and Thiol Compounds in Rich Media Overcome Inhibition of E. coli Growth by CORM-2

**Figure 4:**
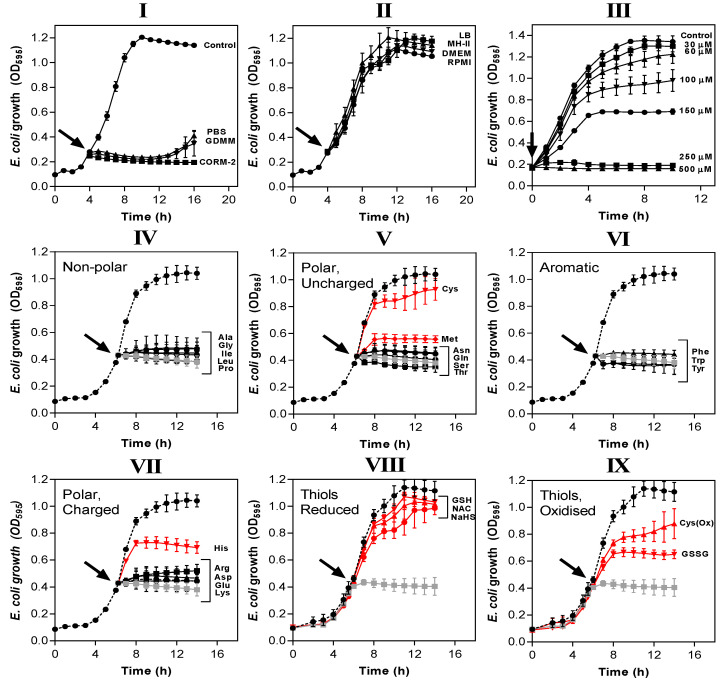
The potency of CORM-2 is influenced by certain compounds added to the growth medium. (**I**,**II**) CORM-2 stocks were prepared by dilution in 10% DMSO/90% H_2_O (‘CORM-2′) or diluted in 10% DMSO/90% medium: (**I**) minimal salts-based media (phosphate-buffered saline, PBS, glucose-defined minimal medium, GDMM) or (**II**) rich nutrient broths (lysogeny broth, LB, Mueller-Hinton II, MH-II, Dulbecco’s modified Eagle medium, DMEM or Roswell Park Memorial Institute medium, RPMI) and left to incubate for 10 min. These stocks were then added at a CORM-2 concentration of 30 µM to *E. coli* grown on GDMM (arrow). Growth (OD_595_) was monitored thereafter. Rich growth media (**II**) protected cells from the growth inhibitory effects of 30 µM CORM-2. In (**III**), cultures grown on GDMM were supplemented with 0.25% (*w*/*v*) casamino acids, and 0–500 µM CORM-2 was added to determine whether a mixture of extracellular amino acids protects against CORM-2-mediated growth inhibition. To determine which specific components of rich media were responsible for protective effects against CORM-2-induced growth inhibition, CORM-2 stocks were mixed with a 2-fold excess of (**IV**) non-polar amino acids, (**V**) polar, uncharged amino acids, (**VI**) aromatic amino acids, (**VII**) polar charged amino acids, (**VIII**) reduced thiol compounds (N-acetyl cysteine, NAC, reduced glutathione, GSH, sodium hydrosulphide, NaHS) or (**IX**) oxidised thiol compounds (cystine, OxCys, oxidised glutathione, GSSG). These stocks were then added to *E. coli* cells grown on GDMM and growth was monitored relative to no-CORM controls (dashed line) and 30 µM CORM-2 prepared by dilution in 10% DMSO/90% H_2_O as standard (grey line). The lines in red indicate amino acids/sulphur compounds that protect against the growth inhibitory effects of 30 µM CORM-2, whereas black lines indicate that the compounds did not exert protective effects. All data are representative of 3–4 biological repeats ± SD.

Amino acids and peptides are major components of rich nutrient broths. Supplementation of GDMM with only 0.25% (*w*/*v*) casamino acids (a casein hydrolysate) dramatically reduced the potency of CORM-2 against *E. coli* so that total inhibition of growth now required 250 µM CORM-2 (Figure 4III). Next, we identified which specific amino acids were responsible for such effects (Figure 4IV–VII). As before, *E. coli* cells were grown to early exponential phase in GDMM prior to addition of 30 µM CORM-2 that had been pre-incubated for 10 min with each amino acid. A two-fold excess of exogenous Cys, Met or His provided significant protection against the growth inhibitory effects of CORM-2 (Figure 4V,VII). Thiol-containing cysteine exhibited the most complete growth protection and so we investigated whether a two-fold excess of other sulphur compounds (namely reduced GSH, NAC, sodium hydrosulphide (NaHS), GSSG, cystine (i.e., Cys(Ox)) could also protect cells against 30 µM CORM-2 (Figure 4VIII–IX). As with cysteine, exogenous reduced thiol-containing GSH, NAC and NaHS were sufficient to fully protect cells against CORM-2-induced growth inhibition, whereas oxidised thiol-containing species (GSSG and Cys(Ox)) gave only partial growth protection, similar to His and Met.

### 3.4. Chelation of CORM-2 by Exogenous Cys, His and Met Prevents Accumulation of Bactericidal Ru(II) into E. coli

**Figure 5:**
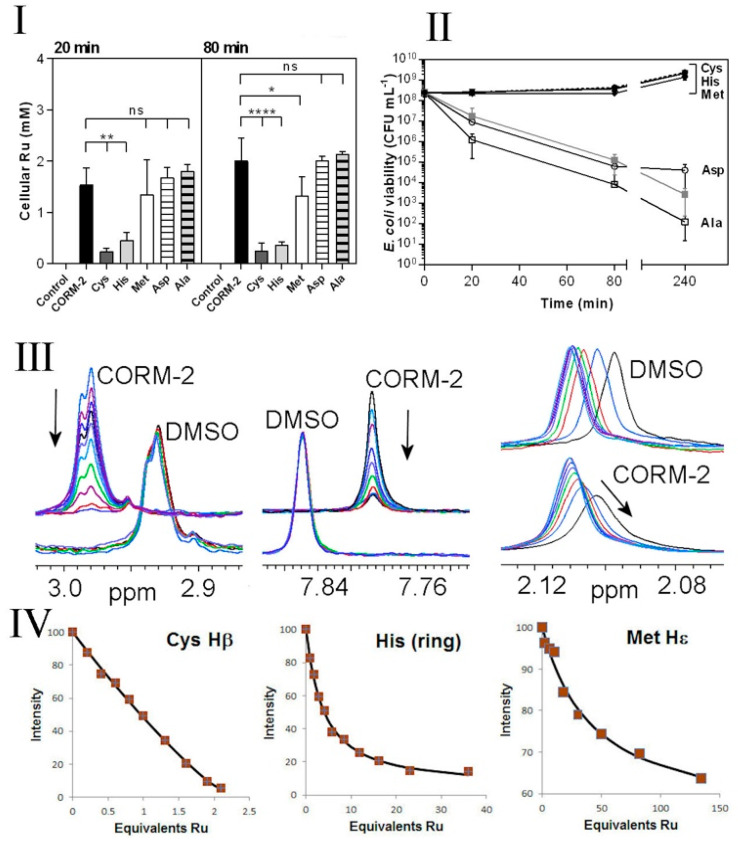
Extracellular amino acids protect *E. coli* against CORM-2, reduce Ru accumulation and in vitro have high affinities for CORM-2. **I**,**II**. Cys, His and, to a lesser extent, Met protect cells against CORM-2 toxicity and prevent intracellular Ru accumulation. CORM-2 stock solutions were incubated with a 2-fold excess of each amino acid prior to addition of CORM-2/amino acid mixtures (30 µM CORM-2, final concentration) to cultures. Extracellular Cys and His significantly reduced the extent of CORM-induced intracellular Ru accumulation (**I**); Cys, His and Met exerted a dramatic reduction in CORM-2-induced cytotoxicity (**II**) (grey line) and restored viability to that of the control (dashed line) (* *p* ≤ 0.05, ** *p* ≤ 0.01, **** *p* ≤ 0.0001, assessed via unpaired t-test). Data in **I** and **II** represent 3 biological repeats, shown as mean ± SD. (**III**,**IV**) Attenuation of toxicity correlates with CORM-2 binding affinities for synthetic peptides containing Cys, His or Met as assessed via ^1^H NMR. The nature of the interactions between CORM-2 and these amino acids was assessed by ^1^H NMR titrations of synthetic peptides (A_3_XA_3_) with CORM-2 or solvent (10% *d*_6_-DMSO/90% D_2_O), where X = Cys (C), His (H) or Met (M). (**III left**) Overlaid ^1^H NMR spectra of A_3_CA_3_ (78.5 μM), showing the Cys Hβ signal titrated with 0–82.4 μM CORM-2 or equivalent volume of solvent. (**III middle**) Overlaid ^1^H NMR spectra of A_3_HA_3_ 125 μM showing the histidine imidazole ring protons titrated with 0–500 μM CORM-2 or equivalent volume of solvent. (**III right**) Overlaid ^1^H NMR spectra of A_3_MA_3_ peptide (78.5 μM) showing the Met Hε protons titrated with 0–4690 μM CORM-2 or equivalent volume of solvent. In each titration the arrow shows the direction of increasing [CORM-2]. (**IV**) Binding curves of the decrease in intensity of ^1^H NMR signals corresponding to Cys, His or Met (as shown in **III**) upon increasing additions of CORM-2. The estimated *K*_d_ of CORM-2 to each peptide was determined to be: 0.3 ± 1 μM for Cys, 130 ± 25 μM for His, 4700 ± 1000 μM for Met. Titrations were performed in 30 mM KPi buffer pH 7.4 and pH was maintained by adjustment with KOH throughout the experiment.

Chelation of non-essential antimicrobial metal ions (e.g., Ag(I)) by exogenous amino acids, peptides or ions within the extracellular milieu is known to reduce their antimicrobial effects by preventing access of the metal ion to metal-sensitive intracellular targets [39]. To investigate whether the protective effects of exogenous Cys, His and Met against CORM-2 could be due to chelation of the compound by these amino acids, we investigated cellular Ru(II) accumulation in *E. coli* cells exposed to 30 µM CORM-2 that had been pre-incubated with a two-fold excess of water (control), Cys, His, Met, Asp or Ala (Figure 5I). At 20 and 80 min after CORM addition, exogenous Cys and His dramatically reduced cellular Ru accumulation relative to CORM only-treated cells (Figure 5I). As a result, cells exposed to a combination of CORM-2 + Cys or CORM-2 + His showed no significant loss of culture viability relative to the non-treated control (Figure 5II). A two-fold excess of Asp or Ala did not prevent Ru accumulation (Figure 5I) and, as expected, did not prevent loss of culture viability in response to CORM-2 (Figure 5II). Interestingly, although a two-fold excess of Met prevented loss of culture viability (Figure 5II), the reduction in cellular accumulation of Ru was minimal (Figure 5I). Taken together, it appears that exogenous Cys and His prevent CORM-2-derived Ru accumulation and thus act as extracellular chelating agents to protect cells against CORM-2-related metal toxicity, whereas exogenous Met may act via a different mechanism.

### 3.5. CORM-2 Binds with High Affinity to Cys, His and to a Lesser Extent Met as Investigated by ^1^H NMR

In order to characterise direct interactions of amino acid sidechains with CORM-2, NMR titrations were undertaken, titrating CORM-2 into solutions of N- and C-terminally blocked peptides AAAXAAA, where X is Cys, His, Met, Asp or Ala. Because CORM-2 is made up as a stock solution in DMSO and subsequently diluted in water before use, control titrations were carried out using the same concentrations of DMSO but without CORM-2 present (i.e., adding 10% DMSO in water). Addition to the cysteine-containing peptide produced a loss of signal intensity from the cysteine sidechains (Figure 5III), with an increase in intensity of several new signals. The nature of these species may warrant further study but is beyond the scope of the present objectives. This implies a slow dissociation rate of the bound CORM-2-derived ligand from the cysteine, and also implies that there is more than one bound species. This was expected, based not only on the different possible Ru-based ligands indicated in Figure 1, but also on the complex solution chemistry of ruthenium carbonyl complexes in water. We showed previously [27] that CORM-3 reacts with phosphate buffer, and also undergoes attack of hydroxide ions on the CO ligands to produce bound carboxylate species, known as the Water Gas Shift reaction [40]. One would expect similar solution chemistry for CORM-2. The signal intensity can be fitted to a standard binding isotherm [35] to produce a fitted dissociation constant of 0.3 μM. There is a large fractional error on this value, because the affinity is strong (the *K_d_* for the ligand is similar to the concentration of the target) and towards the extremity of what can be reliably fitted by NMR, but clearly cysteine binds tightly to CORM-2. Saturation occurs at a ratio of two Ru to one cysteine, i.e., both ruthenium atoms are available for binding. 

Titrations with the other peptides show progressively weaker binding for histidine and methionine, with slow exchange for His and intermediate exchange for methionine, in line with their weaker affinities (Figure 5IV). Peptides containing aspartate or alanine showed changes indistinguishable from control DMSO titrations (Figure 5III). The measured binding affinities are therefore entirely consistent with the observed effectiveness of each of the amino acids on Ru accumulation (Figure 5I) and toxicity (Figure 5II), namely Cys >> His > Met, with no effect for Asp or Ala.

### 3.6. Glutathione Is a Key Extracellular Reactant with CORM-2 and an Intracellular Target of CORM-2-Derived Ru(II)

**Figure 6:**
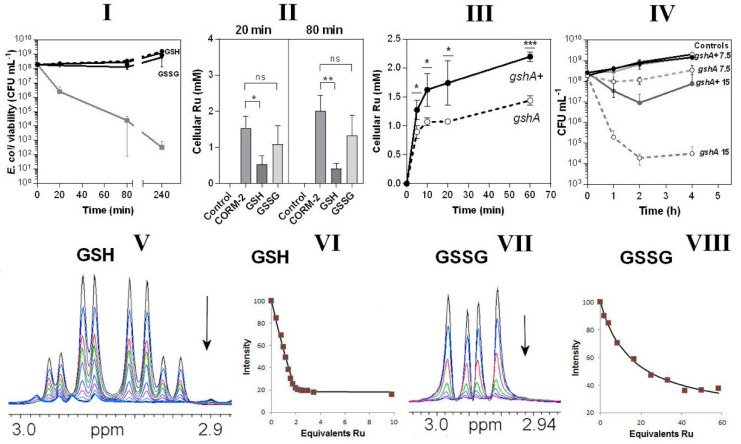
Glutathione is an important intracellular and extracellular target of CORM-2 (**I**–**IV**) and CORM-2 has a much higher affinity for reduced glutathione (GSH) than oxidised glutathione (GSSG) as investigated by ^1^H NMR (V–VIII). In **I**, the effects are shown of extracellular reduced glutathione (GSH) or oxidised glutathione (GSSG). The grey line shows cell viability with CORM-2 alone. GSH fully restored viability to that of the no-CORM control (dashed line). **II** shows CORM-induced intracellular Ru accumulation in the absence or presence of GSH or GSSG (* *p* ≤ 0.05, ** *p* ≤ 0.01, assessed via unpaired t-test). CORM-2 stocks were incubated with a 2-fold excess of the compound prior to addition of 30 µM CORM-2. (**III**) Uptake of CORM-2-derived Ru is significantly reduced in a glutathione-deficient mutant (*gshA*, open symbols, dashed line) compared to the GshA^+^ wild-type parent strain (*gshA*^+^, solid line) (* *p* ≤ 0.05, *** *p* ≤ 0.001, assessed via unpaired *t*-test). (**IV**) Culture viability (CFU mL^−1^) of the glutathione-deficient strain compared to the parent strain following addition of 7.5 or 15 µM CORM-2. Data in **I**–**IV** represent 3 biological repeats ± SD. (**V**) NMR spectra of the Cys Hβ resonances of GSH (1 mM) on titration with 0–5 mM CORM-2 and (**VI**) corresponding binding curve. (**VII**) NMR spectra of the Cys Hβ resonances of GSSG (0.5 mM) on titration with 0–14.5 mM CORM-2 and (**VIII**) corresponding binding curve. In each titration the arrow shows the direction of increasing [CORM-2]. The estimated *K*_d_ of CORM-2 to GSH was determined to be: 2 ± 1 μM for GSH (VI) and 25 ± 5 mM for GSSG (**VIII**). Titrations were performed in 30 mM KPi buffer pH 7.4 and pH was maintained by adjustment with KOH throughout the experiment.

In view of the CORM-2-binding propensity of thiols (Figure 5) and the abundance in cells of the thiol tripeptide GSH [41], we tested the protective effects in bacterial cultures of GSH and oxidised glutathione, GSSG. When included in growing cultures, GSH, and to a slightly lesser extent GSSG, fully protected cells from CORM-2 toxicity (Figure 6I). By pre-incubating the CORM-2 stock solution with these agents it was quantitatively confirmed that GSH was more effective than GSSG in preventing uptake of Ru (Figure 6II). A GSH-deficient mutant (*gshA*) also accumulated significantly less Ru than an isogenic parent strain (Figure 6III) and was hypersensitive to CORM-2 when added to growing cultures (Figure 6IV); the basis of this diminished Ru accumulation is not known and there is no evidence that GSH facilitates Ru compound transport (see Discussion). However, any activity inside a cell that removes a transported substrate will enhance its uptake simply by removing the product of transport. Thus, the observation that lowering the internal concentration of GSH is accompanied by diminution of Ru uptake is consistent with GSH being one of the internal targets for ruthenium. Ru uptake is not abolished because, as we show, there are many other targets additional to GSH. Collectively, these data show the importance of the natural thiol GSH in modulating CORM-2 toxicity. We have not shown that glutathione is the sole intracellular target of CORM but GSH is the most abundant intracellular thiol, reaching c. 10 mM (e.g., [42]).

As expected, the Ru ion of CORM-2 bound tightly to GSH (Figure 6V,VI *K_d_* ≈ 2 μM) but much more weakly to GSSG (Figure 6VII,VIII, *K_d_* ≈ 25 mM). The difference in these NMR titrations confirms the importance of the free thiol group in GSH in the reaction with CORM-2.

### 3.7. CORM-2-Derived Ru(II) Has Multiple Membrane and Intracellular Targets in E. coli Cells

**Figure 7:**
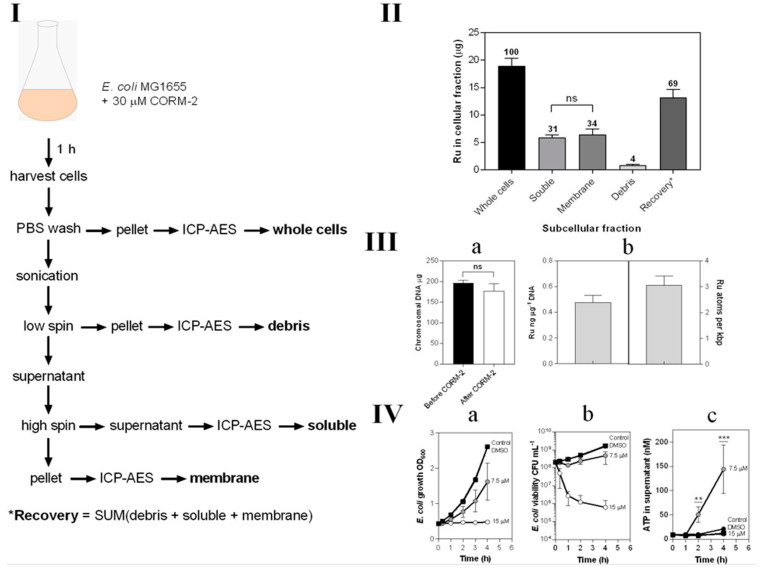
Subcellular distribution of CORM-2-derived Ru(II). (**I**) Scheme demonstrating the process for preparation of subcellular fractions of *E. coli* for quantification of Ru content by ICP-AES. (**II**) The Ru content of each subcellular fraction displaying the percentage of total Ru recovered in each fraction by ICP-AES. (**III**) Localisation of CORM-2-derived Ru on *E. coli* chromosomal DNA. (**IIIa**) the amount of chromosomal DNA recovered from 20 mL *E. coli* cell cultures before (black bar) and after 1 h incubation with 30 µM CORM-2 (white bar). (**IIIb**) the amount of Ru (ng) detected per µg of DNA as determined by ICP-AES of chromosomal DNA samples from cells treated for 1 h with 30 µM CORM-2 and the amount of atoms of Ru per kbp of DNA. (**IV**) Sub-toxic doses of CORM-2 cause ATP leakage from *E. coli* cells. The extent of growth inhibition (**IVa**) and loss in cellular viability (**IVb**) upon addition of 0–15 µM CORM-2 (t = 0 h) to *E. coli* cultures grown on GDMM. (**IVc**) Amount of ATP (nM) detected in the supernatant of *E. coli* cells upon exposure to no CORM, DMSO or sub-toxic (7.5 µM) or toxic (15 µM) levels of CORM-2 (** = *p* ≤ 0.01, *** = *p* ≤ 0.001). All data in **II**–**IV** are representative of 3 biological repeats ± SD. Significance differences were assessed via unpaired t-tests.

Ruthenium is not a naturally occurring element in biology and its uptake is therefore readily and sensitively assayed by ICP-AES (Figure 7). We exposed growing cultures to 30 μM CORM-2 and prepared crude subcellular fractions, namely cytoplasm (‘soluble’), total cell membranes and debris from the breakage and fractionation procedures (Figure 7I). The metal ion was found to be equally distributed between cytoplasm and membranes (Figure 7II). Chromosomal DNA bound almost three atoms of Ru per 1000 bp (Figure 7III). 

In light of the binding of Ru to membranes, we hypothesised that the acute toxicity of CORM-2 might be attributable to membrane damage, leading ultimately to cell lysis. To assay lysis, we measured leakage of ATP from bacteria treated with CORM-2 at sub-toxic and toxic concentrations, exploiting the fact that ATP is generated only in the cytoplasm and not normally exported [43]. A CORM-2 concentration of 15 μM was highly toxic as judged by cessation of growth (Figure 7(IVa)) and rapid loss of cell viability (Figure 7(IVa,IVb)). However, 7.5 μM CORM-2 was only slightly inhibitory to growth (Figure 7(IVa)) and was without major effects on cell viability (Figure 7(IVa,IVb)). Nevertheless, 7.5 μM CORM-2 elicited substantial leakage of ATP from inside cells, indicating extensive cell lysis (Figure 7(IVc)). Surprisingly, 15 μM elicited less ATP leakage. One possibility is that lower CORM-2 concentrations allow continued cellular metabolism while poisoning cells and producing membrane damage, but higher CORM-2 concentrations kill cells more rapidly before membrane leakage can occur.

## 4. Discussion

**Figure 8:**
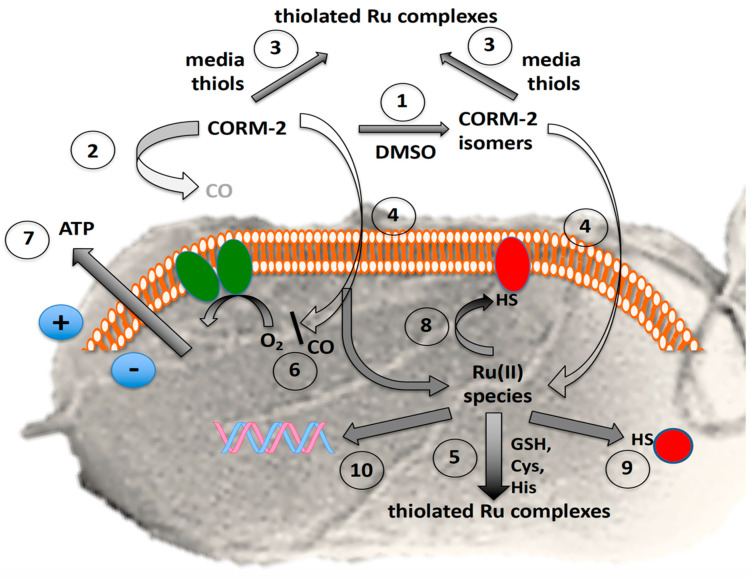
Complexity of the antibacterial actions of CORM-2. The grey background shows the surface view of a bacterial cell with flagella. The orange and white assembly represents phospholipid bilayer in the inner cell membrane containing the respiratory chain (green ovals) and numerous other proteins. The red shapes represent membrane and soluble proteins with exposed -SH groups (exposed histidine imidazole or methionine CH_3_S- are not shown). The blue circles show the charge separation (positive outside, i.e., the protonmotive force) established via respiratory electron transfer or ATP hydrolysis. In the presence of the solvent, DMSO, numerous species are formed by DMSO displacement (1) with low yields of CO, rapidly declining with time (2). Exogenous amino acids and thiols (especially cysteine, glutathione and N-acetyl cysteine) bind CORM-2 and thus protect bacteria from growth inhibition (3); the prevention of toxicity correlates with their CORM-2-binding affinities. CORM-2 or its derived isomers enter cells by unidentified pathways (4). Glutathione and other thiols are intracellular targets of CORM-2 (5). Residual CO inhibits terminal oxidase activity (6) but also may react with transcription factors, Fe–S clusters and other unidentified targets (not shown), leading to loss of membrane integrity and cytoplasmic ATP leakage (7). The fate of intracellular Ru(II) is complex but includes membrane (8) and other intracellular targets (9) targets, including DNA (10).

CORM-3 and CORM-2 are generally considered primarily CO carriers or ‘Trojan Horses’ [33,44,45], delivering a toxic cargo of CO, with the residual Ru ion(s) contributing only a minor role in antimicrobial activity (Figure 8). Other investigators have suggested that antimicrobial activity is due in part to generation of reactive oxygen species, perhaps following respiratory inhibition [46,47]. Recently, a further proposal for CORM-3 toxicity in bacteria has been advanced [48], namely that it elicits intracellular glutamate deficiency and inhibition of nitrogen and tricarboxylic acid cycles. Unlike the present findings, this was attributed to the released CO, but how the gas inhibited glutamate synthesis and iron–sulphur enzymes of the tricarboxylic acid cycle was not established. This conclusion was, however, consistent with the detection of CO within bacteria using the turn-on fluorescent probe COP-1 [49] and the formation of intrabacterial oxidase–CO adducts [23,45], but not readily compatible with the findings from two laboratories that even high CO concentrations in growth media are not toxic [38,50]. Another important unresolved issue in the potential application of CORMs as antimicrobial drugs is why CORM-2 possesses potent antimicrobial activity, yet is reportedly non-toxic to mammalian cells, ex vivo and in whole-animal models, where it exerts therapeutic (including vasodilatory, anti-inflammatory and cardioprotective) effects [19,51,52,53,54].

One reason for the uncertainty in defining CORM-2 actions is the complex speciation of CORM-2 in solvents (Figure 1). Since McLean et al. [26] found that the ‘fast CO release’ from Ru(CO)_3_L_3_ CORMs was due to reaction of the CORM with sulphites, such as sodium dithionite (used as a strong reducing agent in the haemoglobin assay, but much stronger than anything present in vivo), the levels of biologically available CO from CORM-2 or CORM-3 have been unclear. Very little or no CO was detected upon the addition of CORM-2 stocks to phosphate buffers or various growth media, in the absence of sodium dithionite, when analysed via diverse methods. An interesting recent report [55] notes slow release of CO from liposomal CORM-2; however, the CO was detected using the myoglobin assay in the presence of dithionite, and therefore it remains unclear whether the therapeutic benefit was derived from CO release. 

When CO release from CORM-2 was directly measured by FTIR in a sealed solution of 100% DMSO, a maximum of up to 0.4 mol of CO was released from CORM-2 [21], implying that just 40% of CORM-2 dimer (Figure 1(**1**)) dissociated into species **3a** and **3b** and was able to release CO. Rather, Seixas et al. found that approximately 1.8 mol of CO_2_ per CORM could be detected in DMSO/aqueous solutions of CORM-2 [22]. This presumably occurs via water–gas shift chemistry via attack of ^−^OH on CO ligands of **2**, **3a** and **3b,** and is predicted to occur for CORM-3. 

However, in the present study, we found that a maximum of 0.1 mol of CO per mol CORM-2 dimer was available to bind oxyHb (Figure 2II). Even using Ngb, which has been proposed as a useful ligand trap antidote for CO poisoning [29,56], we detected only 6–7% of the CO potentially available from CORM-2 as the carboxyneuroglobin. The apparent discrepancy between our findings and those of Klein [21] may be due to the different methods to assay CO release, namely FTIR [21] and the oxyHb and oxyNgb assays (this work). In our experiments, it is possible that some CO was lost to the atmosphere as the CORM-2 stocks were added to globin solutions. Our conclusion is that, although the exact mechanisms of CO release from CORM-2 are poorly defined quantitatively, it is clear that only negligible amounts of CO are released in solution from CORM-2 in biological experiments, even with such a high-affinity CO trap as Ngb. Only when harsh sulphite-based reductants such as sodium dithionite are present is significant CO detectable. Recently, we showed that CORM-3, Ru(CO)_3_Cl(glycinate), reacts with constituents of biological growth media and buffers, resulting in the creation of numerous Ru(II)–carbonyl species that do not release CO, even upon addition of dithionite. The biological actions of CORM-3 against both bacterial and mammalian cells in vitro were instead shown to be due to the reactivity of the Ru(II) ion, indicating that the designation of Ru(CO)_3_Cl(glycinate) as a mere CO donor is misleading [27]. As CORM-3 is a derivative of CORM-2, we decided to investigate whether the numerous reported biological activities of CORM-2 could instead be due to its Ru(II) ions rather than CO. Previously, Nielsen showed that ruthenium in CORM-2, not CO, is the inhibitor of venom procoagulant activity [57,58], while Dong et al. reported that CORM-2 activates cation currents in endothelial cells independently of CO [59]. Similarly, a recent report [60] showed that both active CORM-2 and ‘inactive iCORM-2’ each exerted biological effects such as cyto- and genotoxicity, antioxidant properties and the ability to induce the HO-1 gene. The released CO as well as iCORM-2 were considered responsible for these effects.

The current data support the view that reaction of CORM-2 with sulphur-containing molecules is similar to that observed for CORM-3 [27]. *E. coli* treated with 60 μM CORM-2 plus cysteine or histidine exhibited no significant loss of viability, but were killed in the absence of amino acids. Note that Southam et al. [27] used only a two-fold excess of these amino acids in observing alleviation of CORM-3 toxicity, but a wider range of supplements (cystine, glycine, serine, etc.) had to be used at concentrations up to 50 μM (about 150-fold excess) to observe alleviation of toxicity [48]. In the present work, the prevention of toxicity correlates with the CORM-2-binding affinities of these amino acids as determined by ^1^H-NMR. Since GSH is the predominant thiol in many bacterial cells, it is proposed to be an intracellular and extracellular target of CORM-2, with CORM-2 having a much higher affinity for reduced glutathione (GSH) than oxidised glutathione (GSSG). Of course, any intracellular protein with surface-exposed Cys, His or Met is also a plausible target for CORM-2 and CORM-3. In this context, we note a recent paper [61] that reports reactivity of both CORM-2 and CORM-3 with a range of compounds including GSSG. These experiments used CORMs incubated for at least 2 h in aqueous buffer, which are likely to have lost all CO by the start of the experiments. Furthermore, these experiments were done in vitro and therefore do not contribute directly to understanding the antimicrobial toxicities of these CORMs.

When certain toxic metals are chelated outside the cell (as for Ag(I) [39]), their toxicity is, unsurprisingly, reduced. Similarly, externally added Cys, His, Met or GSH alleviate the toxicity of Ru–CORM-2 by preventing access of the Ru to the inside of the cell (Figure 2 and Figure 4, Figure 5 and Figure 6), but the mechanism by which Ru–CORM-2 enters the cell is unclear, especially since transcriptomic studies [62] have not identified any involvement of transport proteins. We note that the formal positive charges on the Ru(II) entity of CORM-2 are delocalised around an already hydrophobic molecule [63] that is likely to penetrate the membrane by simple diffusion, reinforced by the existence of a pre-existing electrical gradient, inside negative, maintained by respiration and/or ATP hydrolysis [64].

Whatever the mode of CORM transport (revealed in the Ru uptake assays), CO release presumably occurs both outside and within cells. If, however, CORM-2 is applied extracellularly in the presence of NAC, cysteine and other thiols, its antibacterial activity is attenuated, and its uptake is inhibited [65]. If the thiol is assumed not to inhibit CO release extracellularly (and we are unaware of any such evidence) and if the released CO penetrates cells (as it will), then these experiments strongly support the view that it is not CO, but the accumulated Ru, which is antibacterial. This conclusion is fully supported by the fact that CO is not toxic to bacteria [38]. Interestingly, an oral carbon monoxide release system (OCORS) is claimed to provide precise, controlled, tunable and targeted CO delivery for the treatment of gastrointestinal diseases. OCORS is an oral tablet based on sulphite-induced CO release from the CO-releasing molecule 2 (CORM-2) but toxicological assessments are still needed [66].

The thiol–CORM-2 interactions we describe and/or CORM-2 toxicity may well limit its therapeutic value as a CO delivery agent in mammalian systems, where high levels of biological thiols are present. However, an early study [51] showed that neither CORM-2 nor CORM-3 caused cytotoxicity in an in vitro model of lipopolysaccharide (LPS)-stimulated murine macrophages and produced an increase in HO-1 expression and haem oxygenase activity; this effect was completely prevented by the thiol donor N-acetylcysteine. Nevertheless, a more recent and detailed study showed that both CORM-2 and iCORM-2 (the CO-depleted form) induced significant cellular toxicity evident as decreased cell viability, abnormal cell cytology, increased apoptosis and necrosis, cell cycle arrest and reduced mitochondrial enzyme activity. These results [67] show that the ruthenium-based CORM by-product, iCORM-2, is itself cytotoxic and suggest that the accumulation of iCORM-2 would limit clinical applications of the ruthenium-based CORMs. The validity of using iCORM-2 was also dismissed by [68] who showed that it inhibited activity of recombinant cytochrome P-450. These authors also demonstrated that both CORM-2 and CORM-3 elicited a rapid depletion of oxygen in respirometry which was detected in the medium, even when no cells were present [68]. Thus, several recent studies cast doubt on the proposal that CORM-2 acts solely as a CO releaser. 

We note also that the chemical nature of the CO-depleted ‘iCORM-2’ complexes advocated in some studies is obscure, and so we avoided using it in this study. The structure and ligands are unknown. It is possible that more than CO is lost as the crystal structure of the related CORM-3 with lysozyme shows loss of two CO molecules, but attempts to investigate whether iCORM is a mixture of interconverting compounds failed (B. Mann, personal communication). Experimental preparations of CO-depleted CORM-2 appear heterogeneous and to use such preparations would have produced greater uncertainty.

The fate of CORM-2 in vivo is uncertain. Virtually nothing is known of the chemical forms in which Ru exists in vivo, except for extensive knowledge of the cytological stain Ruthenium Red, Ru polypyridyl complexes and anticancer Ru-containing molecules (reviewed in [24]), all of which are chemically unrelated to CORM-2. Based on their mechanism of action, various possibilities may be considered as follows. (i) Structural: The Ru ion(s) have a structural role, i.e., the Ru provides shape to the active compound via a coordination sphere of ligands. These complexes are inert and biologically stable, the Ru interacting with the target only via noncovalent interactions. (ii) Carrier: The Ru ion(s) simply function as carriers for the active drug, most commonly an organic compound. (iii) Functional: The Ru ion(s) have a functional role, i.e., the antimicrobial activity of the compound is mediated by the Ru ion directly coordinating to biological targets. These are relatively biologically unstable compounds, often prodrugs, which contain labile ligands. (iv) Photoactivated: The Ru compound is active only upon illumination where it can act as a photosensitizer. We consider class (iii) to be the most likely mechanism here [24].

However, in the context of CORM-2 interactions in vivo, Cys and GSH are of special interest as CORM-2-reactive molecules, and the liver is regarded as the site of cysteine homeostasis. Much of the sulphur amino acid load reaching the liver is incorporated into GSH and then exported for use by other tissues [69], where it is broken down by tissues that express γ-glutamyl transpeptidase. The release of Cys in the peripheral circulation results in tissues being exposed to relatively high concentrations of cysteine. Hepatic Cys levels in rats are between 0.02–0.04 μmol g^−1^, and for GSH 1.65–4.36 μmol g^−1^, dependent on diet [70]. Cys and cystine constitute the predominant low-molecular-weight thiol–disulphide pool in human plasma [71]. However, GSH is more likely to remain reduced in an oxidative environment than Cys and is maintained in tissues at millimolar concentration with a relatively reduced redox state [72], indicating that the Cys/CySS and GSH/GSSG pool are not in equilibrium [71]. Such high levels of thiols in the body suggest limited access of administered CORM-2 to tissues. Thus, our results suggest that the biological effects of CORM-2 in vivo are due to ruthenium rather than CO release. It is, however, important to note that the results reported here are obtained from *E. coli*, and require experimental confirmation in mammalian systems. 

Whether or not long-term toxicity is confirmed, the present data raise the question: is CORM-2 effective as a CO releaser in vivo? The levels of CORM-2 accumulating in liver are unknown and would be of dubious relevance as CO is likely to have been released before access to the liver. Nevertheless, Seixas et al. [22] measured the ability of CORM-3 to deliver CO specifically to organs and tissues and compared it with CO inhalation by mice. CO accumulated mainly in the liver, kidney and spleen, where CO levels increased by a factor of ca. 3, while that in the heart increased by a factor of 2; the lungs and brain remained essentially at baseline values.

## 5. Conclusions

To summarise, we demonstrated that the amount of CO released from CORM-2 is inadequate to explain its toxicity. Rather, CORM-2 reacts with thiol-containing compounds, of which cysteine (free and in proteins) and reduced glutathione are key players in its physiological effects. These reactions dramatically reduce the toxic effects of CORM-2. While the biological chemistry of Ru complexes is far from completely understood [24], the toxic effects of Ru CORMs on bacterial and (by extrapolation) mammalian cells can now be largely attributed to the chemical reactivity of Ru, and not CO.

## Data Availability

Data are contained within the article.

## Abbreviations

The following non-standard abbreviations are used in this manuscript:

GDMM	glucose-defined minimal medium
Hb	haemoglobin
KPi	potassium phosphate buffer
Mb	myoglobin
Ngb	neuroglobin
CORM	carbon monoxide-releasing molecule
CORM-2	tricarbonyldichlororuthenium(II) dimer, Ru_2_Cl_4_(CO)_6_
CORM-3	Ru(CO)_3_Cl(glycinate)]
iCORM	‘inactive’ CORM
